# Temporal and Spatial Responses of Chinese Red Pandas to Anthropogenic Disturbance in Meigu Dafengding National Nature Reserve, Sichuan

**DOI:** 10.1002/ece3.74208

**Published:** 2026-08-16

**Authors:** Ying Fu, Minghua Chen, Bin Feng, Mengyi Duan, Zhisong Yang, Ke He, Hong Zhou

**Affiliations:** ^1^ Key Laboratory of Southwest China Wildlife Resources Conservation (Ministry of Education) China West Normal University Nanchong Sichuan China; ^2^ College of Giant Panda China West Normal University Nanchong Sichuan China; ^3^ Sichuan Meigu Dafengding National Nature Reserve Administration Liangshang Sichuan China; ^4^ Sichuan Academy of Giant Panda Chengdu Sichuan China

**Keywords:** activity rhythm, *Ailurus styani*, anthropogenic disturbance, occupancy modeling

## Abstract

Global biodiversity decline is increasingly driven by anthropogenic disturbances. This study focuses on the endangered Chinese red panda (*Ailurus styani*) in the Sichuan Meigu Dafengding National Nature Reserve, using an integrated approach that combined kernel density estimation, occupancy modeling, and generalized additive models to systematically investigate the impacts of anthropogenic disturbance on this species. The results showed that the Chinese red panda exhibited a diel activity rhythm with a midday peak, and its seasonal activity intensity was low in spring and high in summer, autumn, and winter. Spatially, the species preferred high‐elevation areas and showed spatial avoidance of cattle (Species Interaction Factor (SIF) = 0.81) and sheep (SIF = 0.60), but co‐occurred with domestic dogs (SIF = 1.53) and horses (SIF = 1.80). Generalized additive model results further confirmed that anthropogenic disturbance had a significant negative impact on the Chinese red panda. These findings provide important insights for the conservation of this species and for understanding wildlife behavioral responses to anthropogenic disturbance.

## Graphical abstract

We explored red panda behavioral adaptations to disturbances from both temporal and spatial perspectives. Red pandas favor high‐altitude areas with minimal disturbance and abundant resources. Findings provide a foundation for developing cohesive protection strategies, crucial for long‐term conservation.

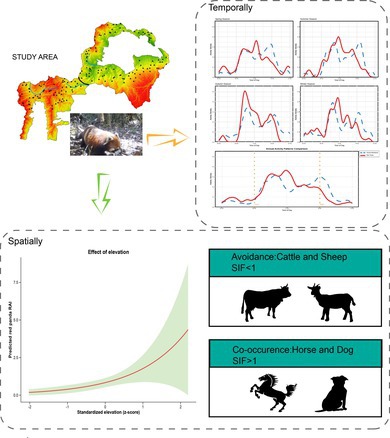

## 1 Introduction

With continuous global population growth and societal development, human‐induced environmental changes pose severe challenges to wildlife. Issues such as habitat loss and fragmentation, climate change, and environmental pollution are becoming increasingly prominent (Laliberte and Ripple 2004; Wei et al. 2014). Facing intensifying anthropogenic disturbances, wildlife often adapt by altering their spatial distribution and behavioral patterns. For instance, livestock activities force giant pandas ( *Ailuropoda melanoleuca* ) to shift toward lower elevations, areas with poorer forest cover, and steeper slopes (Zhang et al. 2017). Human disturbances can also drive wildlife to adopt more nocturnal activity patterns (Ikeda et al. 2022). These behavioral adjustments align with the “risk‐disturbance hypothesis,” which posits that human‐generated disturbances functionally resemble predation risks, prompting animals to make similar trade‐offs (Frid and Dill 2002). Therefore, systematically investigating the spatiotemporal response mechanisms of wildlife to anthropogenic disturbances is critical for developing effective conservation strategies.

In China, the giant panda, a species of great conservation concern, has been intensively studied, and a large body of research has confirmed the negative impacts of anthropogenic disturbance on its habitat. For instance, in the Liangshan Mountains, suitable giant panda habitats significantly overlap with areas affected by livestock grazing and bamboo shoot collection, exerting synergistic effects on habitat suitability and daily activity rhythms (Pu et al. 2024). Similarly, in Wolong Nature Reserve, habitat overlap between horses and giant pandas has been documented, with horses consuming large quantities of bamboo, reducing panda habitat utilization (Hull et al. 2014). However, research on how the sympatric and bamboo‐dependent Chinese red panda (*Ailurus styani*) responds to anthropogenic disturbances remains limited. Recent taxonomic revisions recognize two distinct red panda species: the Himalayan red panda ( *Ailurus fulgens* ) and the Chinese red panda (Hu et al. 2020). The latter is primarily distributed in Sichuan, Yunnan, and eastern Tibet, with Sichuan serving as its core range. Alarmingly, over recent decades, the Chinese red panda has experienced significant contraction and population decline (Wei, Feng, Wang, and Hu 1999). Recent surveys suggest possible regional extinction in much of its former Minshan Mountain range distribution, raising urgent conservation concerns (Thapa et al. 2020; National Forestry and Grassland Administration 2021; Ruan et al. 2024). Habitat selection in red pandas is influenced by two key factors: climate and anthropogenic disturbances. Given the irreversible trend of global warming, assessing the impact of human activities on this species is critical (Li et al. 2025).

Despite these pressing conservation challenges, critical knowledge gaps persist in Chinese red panda research. Current studies primarily focus on habitat suitability assessments and basic activity rhythm analyses. For example, Zhang et al. (2011) only analyzed the activity rhythm of this species in the Fengtongzhai Nature Reserve, revealing a crepuscular activity pattern with significant seasonal differences; on habitat suitability assessment, showing that the area and proportion of suitable habitat in the Xiaoxiangling Mountains were both significantly higher than those in the Daxiangling Mountains (Li et al. 2025). Although these studies provide a foundation for understanding the spatiotemporal behavior and habitat requirements of the Chinese red panda, they have not addressed the species' spatiotemporal dynamic responses to specific anthropogenic disturbances, such as livestock grazing. Therefore, there is currently a lack of research on the spatiotemporal variation of the Chinese red panda under disturbances like grazing.

The Meigu Dafengding National Nature Reserve, situated in the core area of the Liangshan Mountain Range, serves as a critical habitat for the Chinese red panda. However, the reserve is under pressure from livestock grazing, along with illegal wood collection and other forest product extraction, posing the main threats to red panda habitats (Zhou et al. 2013). Despite this pressing conservation challenge, the spatiotemporal adaptation mechanisms of Chinese red pandas to anthropogenic disturbances remain poorly quantified. Therefore, this used camera‐trapping, combined with kernel density estimation and occupancy models, to systematically investigate the spatiotemporal response mechanisms of the Chinese red panda in this reserve to anthropogenic disturbance. By quantifying diel activity overlap and fine‐scale spatial correlations between Chinese red pandas and disturbance sources, this study elucidates behavioral plasticity in niche partitioning across temporal and spatial dimensions. Our findings provide actionable insights for designing targeted conservation strategies that balance ecological integrity with local livelihood needs in human‐influenced landscapes.

## 2 Study Area

**FIGURE 1:**
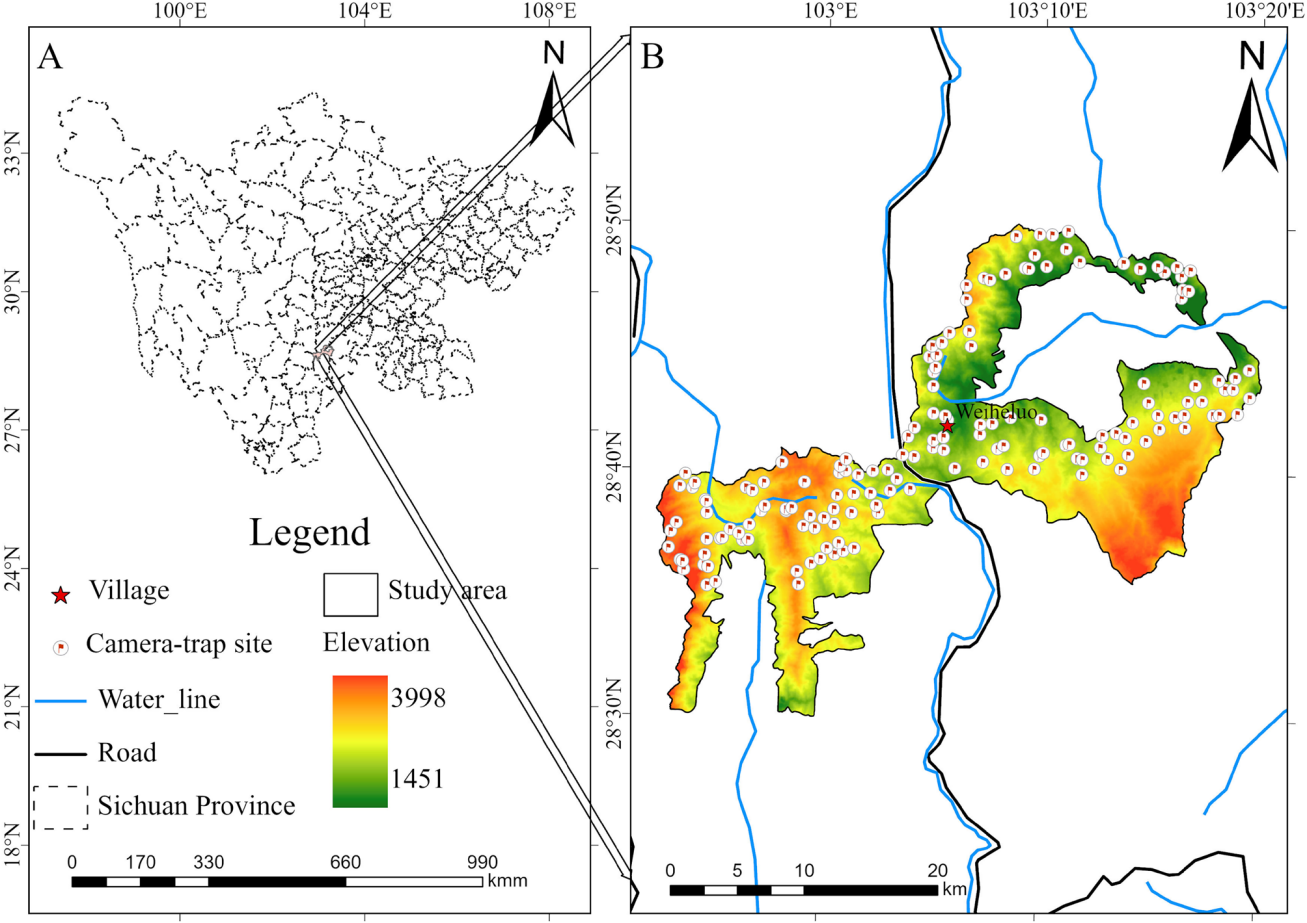
Study area for camera‐trapping survey and environmental characteristics in the Sichuan Meigu Dafengding National Nature Reserve. (A) Sichuan Province county boundary map with a 0–990 km scale bar and legend. Elevation gradients are depicted by red (high: 3998 m) to green (low: 1451 m) colors. Ecological survey elements include: black triangles (camera location), black solid lines (roads), blue solid lines (water line), and green stars (village). This integrated visualization highlights the region's topographical complexity, infrastructure distribution, village, and systematic sampling design. (B) Detailed map of the Meigu Dafengding Reserve, showing a 0–20 km scale bar.

The Sichuan Meigu Dafengding National Nature Reserve (28°30′–28°50′N, 102°52′–103°20′E; hereinafter referred to as the Meigu Dafengding Reserve) is located on the southeastern margin of the Qinghai‐Tibet Plateau, in the northeastern part of Meigu County, Liangshan Yi Autonomous Prefecture, Sichuan Province. Encompassing 506.55 km^2^, the reserve spans an elevational gradient of 1451–3998 m (Figure 1). This region experiences a humid subtropical monsoon climate characterized by low annual temperature variation but high diurnal fluctuations, with a mean annual temperature of 10.2°C and annual precipitation of 1089 mm. Seasons are defined locally as follows: spring (March–May), summer (June–August), autumn (September–October), and winter (November–February). Predominantly inhabited by the Yi ethnic group, surrounding communities practice traditional livestock husbandry relying on free‐ranging cattle ( *Bos taurus* ), goats ( *Capra hircus* ), and horses ( *Equus caballus* ) (Ji 2000). These unrestrained grazing activities within the reserve constitute a pervasive anthropogenic disturbance. The reserve supports endangered mammals including giant panda, Chinese red panda, and forest musk deer ( *Moschus berezovskii* ). Critical bamboo resources comprise *Bashania fangiana*, *Yania ailuropodina*, *Yushania dafengdingensis*, *Yushania brevipaniculata*, *Yushania maculata*, and *Yushania mabianensis*, forming essential forage and habitat components (Shi et al. 2020; Chen et al. 2022).

## 3 Method Details

### 3.1 Survey Data

The study was conducted from January 2023 to April 2024, during which 197 infrared camera sites were established in the Meigu Dafengding Nature Reserve, using Ltl‐5273 infrared cameras (Ltl Acorn, China) (Figure 1). Camera traps were deployed using a combination of systematic grid and habitat‐optimized placement. First, based on the topography and accessibility of the reserve, a grid of approximately 1 km × 1 km was overlaid to cover different elevations and major habitat types, and preset survey units were determined for each grid cell. Within each unit, rather than placing the camera mechanically at the grid center, specific sites with frequent animal activity and good imaging conditions were preferentially selected, such as animal trails, water sources, forest gaps, and areas with concentrated feces or tracks. Sites were also chosen to provide an open field of view and moderate cover to improve the detection probability of target animals. To ensure statistical independence of samples, a minimum linear distance of 500 m was maintained between adjacent cameras. Cameras were uniformly mounted on tree trunks at a height of 0.4–0.6 m above the ground. All cameras were set with identical parameters: 24‐h operation mode, a shooting pattern of “3 consecutive photos + 20‐second video”, sensitivity set to “medium”, and a trigger interval of 1 min. The latitude and longitude of each camera were recorded in detail, and batteries were replaced regularly to maintain long‐term monitoring (Xiao et al. 2014).

The infrared cameras were deployed across an elevational range of approximately 2000–4000 m, divided into four 500‐m elevational zones. The cameras covered six vegetation types: mixed evergreen‐deciduous broadleaf forest, mixed coniferous‐broadleaf forest, coniferous forest, deciduous broadleaf forest, evergreen broadleaf forest, and shrubland (Table S1).

### 3.2 Environmental Variables

Based on similar studies, the environmental factors included seven variables related to topography, vegetation, and anthropogenic disturbance (Vitekere et al. 2020).

1. Topographic factors: Topographic variables included elevation (ELE), slope (SLP), aspect (ASP), and distance to water (DTW). All variables were derived from a 30 × 30 m resolution digital elevation model of Meigu County (https://www.gscloud.cn/) and calculated using ArcGIS 10.8.
2. Enhanced Vegetation Index (EVI): The EVI data were obtained from the Geospatial Data Cloud platform, with a spatial resolution of 250 m and a temporal resolution of 16 days.
3. Anthropogenic disturbance: Distance to road (DTD) and distance to residential area (DTR) were used as disturbance variables and obtained using the Euclidean Distance tool in ArcGIS 10.8. Road and settlement vector data were downloaded from the National Catalog Service for Geographic Information (https://www.webmap.cn/).

### 3.3 Relative Abundance

This study employed the Relative Abundance Index (RAI) and the Time Period Relative Abundance Index (TRAI) to assess species diversity and the relative activity levels of Chinese red pandas and domestic animals (mainly cattle, sheep, horses, and dogs) within the Meigu Dafengding Nature Reserve. In accordance with camera‐trapping protocols, all consecutive photo or video records of the same species within a 30‐min interval were considered a single independent event (O'Brien et al. 2003). The RAI is defined as the number of independent events for a given species per unit of survey effort and serves as a key metric for estimating relative population abundance using camera‐trap data. It is calculated as follows:

$$\mathrm{RAI}=\left(N_{i}/D\right)\times 1000$$

where $N_{i}$ represents the total number of independent effective photo captures of species *i* across all infrared camera locations, and *D* denotes the sum of working days for all infrared camera sites.

TRAI was used to quantify species' relative activity levels during specific time periods (Chen et al. 2024), calculated as follows:

$$\text{TRAI}=\left(P_{t}/D\right)\times 100$$

where $P_{t}$ represents the number of independent events of a certain species within the time period *t*, and *N* denotes the total number of independent events of all species within the same time period.

To investigate the effects of anthropogenic disturbance on red panda distribution, we grouped yak ( *Bos grunniens* ) and yellow cattle ( *Bos taurus* ) into a single category (“cattle”) and goats and sheep ( *Ovis aries* ) into another (“sheep”). Cattle, sheep, horses, and dogs ( *Canis lupus familiaris* ) were subsequently defined as anthropogenic disturbance indicators. The relative abundance index essentially reflects the relative detection rate or encounter rate of a species, rather than true population abundance. The RAI of red pandas and anthropogenic disturbance was calculated for each camera station.

In summary, the RAI is influenced by factors such as camera‐trap placement, differences in detection probability among species and individuals, and animal activity rhythms. Therefore, when using RAI as a proxy for relative population size, the results should be interpreted with caution.

### 3.4 Activity Rhythm

Kernel density estimation was employed to characterize both seasonal and annual activity patterns of the species (Ridout and Linkie 2009). Seasonal activity rhythms were defined according to the climatic characteristics of Meigu Dafengding Nature Reserve: spring (March–May) represents the vegetation recovery period and a potential breeding period for the Chinese red panda; summer (June–August) is characterized by high temperatures and abundant resources; autumn (September–October) is a critical period for the Chinese red panda to acquire energy reserves before winter; and winter (November to the following February) imposes resource constraints and thermoregulatory challenges (Wei, Feng, Wang, and Li 1999; Zhang et al. 2009; Zeng et al. 2023).

The coefficient of overlap (Δ) was used to quantify the degree of temporal overlap in diel activity patterns between Chinese red pandas and anthropogenic disturbances. Values of Δ range from 0 to 1, where values closer to 1 indicate higher overlap and values closer to 0 indicate lower overlap (Linkie and Ridout 2011). Following conventional interpretation, Δ < 0.5 was considered low overlap, 0.5 ≤ Δ < 0.7 moderate overlap, and Δ ≥ 0.7 high overlap. All analyses were conducted in R version 4.5.0 (R Core Team 2006). Specifically, kernel density estimation and calculation of the overlap coefficient were performed using the overlap package, with 95% confidence intervals derived via smoothed bootstrap sampling (10,000 iterations) (Linkie and Ridout 2011).

### 3.5 The Single‐Season Single‐Species Occupancy Model

Occupancy modeling provides a flexible analytical framework for assessing species habitat preferences. This study used single‐season single‐species occupancy models to analyze the influence of environmental factors on species distribution rates (MacKenzie et al. 2002). To ensure that the occupancy model could effectively distinguish true absence from false absence, we first constructed a detection history matrix for the target species and filtered the camera‐trap sites. Sites with fewer than 30 trap‐days (i.e., days the camera was operational) and no detection of the target species were excluded. This filtering substantially improved the robustness and accuracy of parameter estimates without sacrificing detection information from confirmed occupied sites. After site selection, detection histories for the study species were compiled using 5‐day sampling occasions, resulting in 37 occasions over the study period. Sites with an insufficient number of sampling occasions were removed, and 161 of the initial 197 sites were retained for final analysis (Xiao et al. 2019). In occupancy modeling, the term “season” does not necessarily refer to a geographical season but denotes the temporal period during which data were collected (MacKenzie et al. 2006). Based on the assumptions of single‐season single‐species occupancy models (Shannon et al. 2014), this study used monitoring data from the warm season (May–October 2023) for model construction. Selecting the warm season aligns with the model's closure assumption, enables monitoring during peak human activity periods, and allows observation of predator behavior during high wildlife activity (Xiao et al. 2019; Jachowski et al. 2020).

Environmental variable layers imported into ArcGIS 10.8 were extracted to sampling points using the “Extract Values to Points” tool. Spearman correlation analysis was conducted among all environmental variables. When the absolute correlation coefficient |*r*| > 0.7 between any two variables, models containing both variables were excluded to avoid multicollinearity (Figure S1). Finally, occupancy modeling was conducted using the ‘unmarked’ package in R (Xiao et al. 2019). Models were ranked based on Akaike's information criterion (AIC), and models with ΔAIC ≤ 2 were considered the top models. When multiple top models were identified, model‐averaging was applied to synthesize the results (Burnham and Anderson 2002). An environmental variable was considered to have a statistically significant effect on occupancy probability when *p* < 0.05. Subsequently, accounting for imperfect detection, we calculated the predicted occupancy probability (Ψ, psi) by setting all covariates to their mean values in the logistic regression model (MacKenzie et al. 2002).

### 3.6 The Dual‐Species Occupancy Model

In this study, two‐species occupancy models were used to examine the effects of anthropogenic disturbance (cattle, dogs, horses, and sheep) on the Chinese red panda. The model includes eight parameters and requires the specification of a dominant species and a subordinate species (Table S3) (Richmond et al. 2010). Based on the model assumptions, the anthropogenic disturbance sources (cattle, dogs, horses, and sheep) were designated as the dominant species (Species A), and the Chinese red panda as the subordinate species (Species B). Previous research has shown that agricultural land‐use change is considered the greatest threat, affecting 85% of threatened small carnivore species (Marneweck et al. 2022). In this study, the recorded activities of cattle, sheep, and horses represent livestock grazing disturbance, while domestic dogs represent an invasive species; spatially, these animals constitute a dominant factor that the Chinese red panda needs to actively avoid. Therefore, treating anthropogenic disturbance as the dominant species and the Chinese red panda as the subordinate species is consistent with both the model assumptions and the ecological reality. To avoid model non‐convergence, the probability of detecting species B was assumed to be unaffected by the detection of species A at the same site (rBA = rBa) (Wang et al. 2015).

For the model analysis, single‐species occupancy models were first used to simplify candidate model sets. The occupancy covariates selected from the best‐performing single‐species model for each species were then carried forward as the optimal covariates for niche analysis in the two‐species occupancy model framework. Following previous research (Murphy et al. 2019), eight candidate models were constructed for each species (Table S4). Models were ranked using Akaike's information criterion (AIC), and the model with the lowest AIC value was selected as the best model. Parameters were extracted from this model to calculate the Species Interaction Factor (SIF) estimate. SIF values were interpreted as follows: SIF = 1 indicates independent spatial distributions between the two species; SIF < 1 suggests spatial segregation; and SIF > 1 implies spatial overlap (Richmond et al. 2010). All two‐species occupancy models were implemented using Presence software (version 2.13.47) (MacKenzie et al. 2017).

### 3.7 General Additive Model Analysis

To further quantify the effects of anthropogenic disturbance on Chinese red panda distribution and its relationship with elevation, this study employed Generalized Additive Models (GAMs) based on the Tweedie distribution. The Tweedie distribution, part of the exponential dispersion family, is particularly effective for handling data structures containing a substantial proportion of zeros alongside continuous positive response values (Dunn and Smyth 2008). This distribution has proven to be a valuable tool for analyzing such data in various fields, including ecology and actuarial science (Duan et al. 2020). GAMs are well‐suited for capturing complex nonlinear relationships between predictor and response variables and have been widely applied in ecological data analysis (Wood 2017; Guisan et al. 2002).

To maintain consistency with the occupancy model analysis, this study focused on warm‐season data, using climatic variables for the warmest quarter (Mean Temperature of Warmest Quarter, Bio10; Precipitation of Warmest Quarter, Bio18) to characterize climatic conditions. The model used the RAI of the Chinese red panda calculated at the site scale as the response variable, with anthropogenic disturbance RAI as the core predictor, while controlling for the effects of elevation, Enhanced Vegetation Index (EVI), vegetation type, distance to road, distance to residential area, distance to water, and key climate variables. After the predictor variables were standardized, smoothing parameters were estimated using restricted maximum likelihood (REML) (Wood 2011), and model diagnostics were performed to ensure convergence and goodness‐of‐fit. This analytical approach was designed to test the following hypotheses: (1) anthropogenic disturbance has a significant negative effect on Chinese red pandas; (2) after controlling for anthropogenic disturbance, Chinese red pandas still exhibit a preference for higher elevation areas; and (3) climatic conditions exert an independent influence on Chinese red panda distribution. All analyses were conducted in R version 4.5.0, using the mgcv package for model fitting and diagnostics (Wood 2017).

## 4 Results

### 4.1 Relative Abundance

From 2023 to 2024, a total of 197 infrared cameras were retrieved, accumulating 44,606 camera trap days of monitoring. During the entire monitoring period, all photos or videos of the same species captured within a 30‐min interval were defined as one effective photo. Anthropogenic disturbances were quantified across four categories: cattle with 737 detections (RAI = 16.52), sheep with 130 detections (RAI = 2.91), horses with 240 detections (RAI = 5.38), and domestic dogs with 26 detections (RAI = 0.58) (Table 1, Table S2).

**TABLE 1:** Number of effective photos and RAI of the Chinese red panda and anthropogenic disturbance.

Species	Total number of valid photos	Relative abundance
Chinese red panda	284	6.37
Cattle	737	16.52
Sheep	130	2.91
Horse	240	5.38
Dog	26	0.58
Total	1417	

**FIGURE 2:**
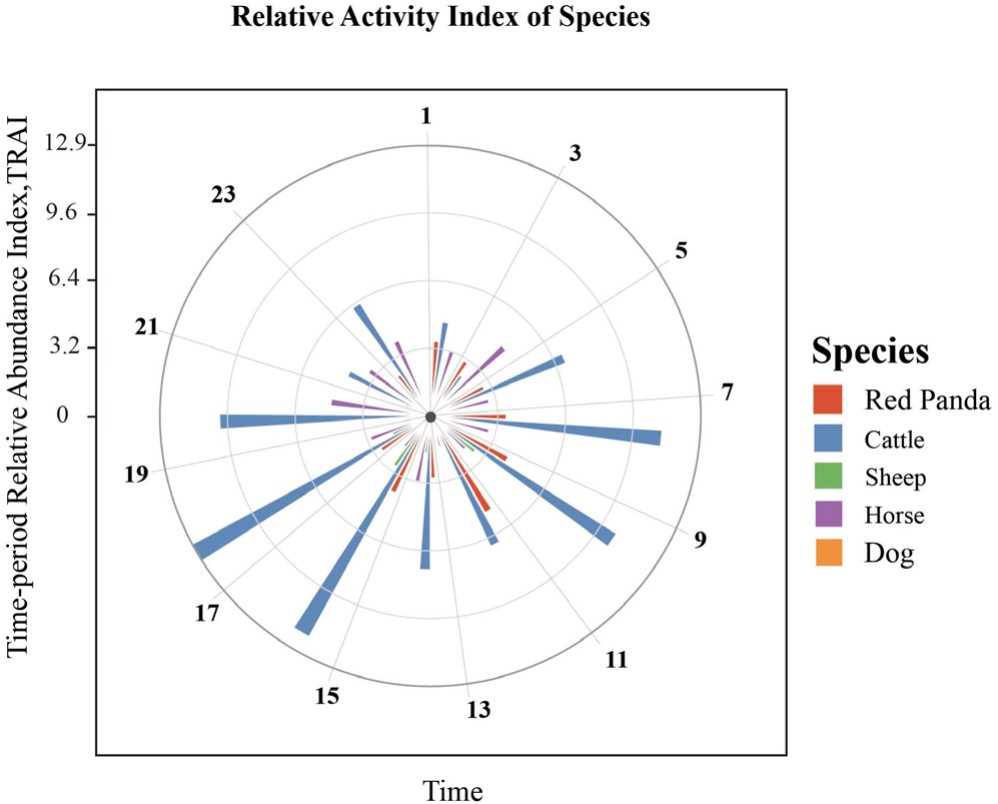
TRAI of the Chinese red panda, cattle, sheep, horse, and dog across different time periods. The angular axis represents time of day (h), with labels at 2‐h intervals (i.e., 1, 3, 5, …, 23 h). The radial axis indicates the TRAI value, reflecting the activity intensity of each species at different times. Species are color‐coded as follows: Chinese red panda (red), cattle (blue), sheep (green), horses (purple), and dogs (orange).

TRAI analyses revealed significant diel activity pattern differences between Chinese red panda and domestic animals (Figure 2). Chinese red pandas exhibited temporally dispersed activity with relatively low overall intensity, showing a single activity peak between 11:00 and 12:00. In contrast, cattle, sheep, and horses all exhibited a crepuscular activity pattern, with activity concentrated mainly between 04:00–10:00 and 15:00–21:00. Among them, cattle were particularly active during 15:00–18:00. Domestic dogs showed no discernible diel pattern due to limited detections (*n* = 26) (Table 2).

**TABLE 2:** Number of valid photos of Chinese red panda under anthropogenic disturbance in different time periods (S: species).

Time periods	Chinese red panda	Cattle	Sheep	Horse	Dog
01:00 ≤ S < 03:00	22	28	4	20	0
03:00 ≤ S < 05:00	9	7	1	14	0
05:00 ≤ S < 07:00	12	29	4	12	0
07:00 ≤ S < 09:00	34	104	11	27	1
09:00 ≤ S < 11:00	48	120	31	26	8
11:00 ≤ S < 13:00	55	71	9	16	5
13:00 ≤ S < 15:00	28	69	17	30	4
15:00 ≤ S < 17:00	32	95	23	15	4
17:00 ≤ S < 19:00	27	123	19	29	2
19:00 ≤ S < 21:00	7	61	7	29	1
21:00 ≤ S < 23:00	5	17	3	14	1
23:00 ≤ S < 01:00	5	13	1	8	0

### 4.2 The Chinese Red Panda Activity Rhythm

**FIGURE 3:**
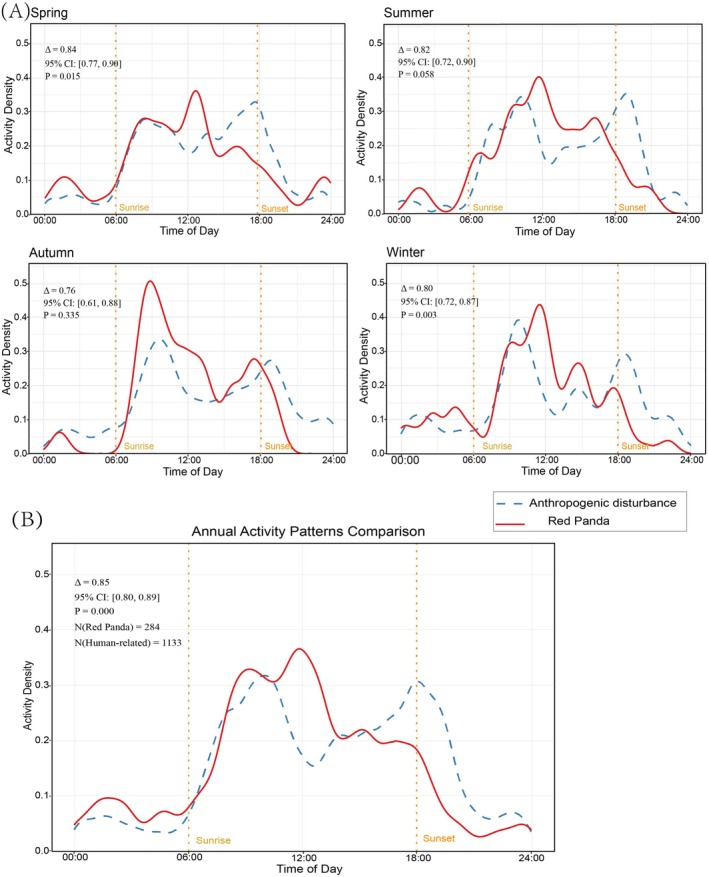
Seasonal and annual diel activity rhythms of the Chinese red panda and anthropogenic disturbance in the Sichuan Meigu Dafengding National Nature Reserve. (A) Seasonal diel activity rhythms. Subpanels show spring, summer, autumn, and winter activity density (*y*‐axis) over time (*x*‐axis, 00:00–24:00) for Chinese red pandas (red solid line) and anthropogenic disturbance (blue dashed line) in the Meigu Dafengding National Nature Reserve, Sichuan, China. Each subpanel displays the activity overlap coefficient (Δ), 95% confidence interval (95% CI), and significance (*p*‐value). Orange dashed vertical lines indicate sunrise and sunset times, highlighting patterns of overlap and divergence between the two activity rhythms across seasons. (B) Annual diel activity rhythm. Activity density (*y*‐axis) over time (*x*‐axis, 00:00–24:00) for Chinese red pandas (red solid line) and anthropogenic disturbance (blue dashed line) on an annual scale. Displays the activity overlap coefficient (Δ), 95% confidence interval (CI), significance (*p*‐value), and sample sizes (*N*). Orange dashed vertical lines indicate sunrise and sunset times, illustrating overall differences in activity rhythms between the two across the year.

The results of seasonal activity rhythm analysis showed that the activity peaks of the Chinese red panda and anthropogenic disturbance differed across seasons (Figure 3A). The Chinese red panda's activity peak occurred around 12:00 in spring, summer, and winter, whereas in autumn it exhibited a distinct bimodal pattern, with peaks at around 09:00 in the morning and around 18:00 in the evening. In contrast, peaks of anthropogenic disturbance activity occurred at 08:00–09:00 and 17:00–18:00 during spring. Summer disturbance was concentrated between 08:00–11:00 and 18:00–20:00. Autumn disturbance peaked at 09:00–10:00 and 18:00–20:00. Winter disturbance was concentrated at 09:00–10:00 and 18:00–19:00. Kernel density analysis further revealed high overlap in activity patterns between Chinese red pandas and anthropogenic disturbance across all seasons. Overlap was highest in spring (coefficient of overlap Δ4 = 0.84, 95% CI [0.77–0.91], *p* = 0.015). Winter also showed significant overlap (Δ4 = 0.80, 95% CI [0.72–0.87], *p* = 0.005). Although the summer overlap coefficient was high (Δ1 = 0.82, 95% CI [0.72–0.90]), statistical significance was marginal (*p* = 0.068). Autumn overlap was 0.76 (95% CI [0.62–0.88]), but statistical significance was not achieved (*p* = 0.305), likely due to sample size limitations (Table S5).

On an annual scale, Chinese red pandas exhibited a predominantly diurnal activity pattern, with activity peaking between 9:00 and 12:00, whereas anthropogenic disturbance displayed a crepuscular‐type activity pattern, concentrated around 10:00 and 18:00 (Figure 3B). The annual activity overlap between the two reached 0.85 (95% CI [0.80–0.89], *p* < 0.05), indicating high temporal overlap.

### 4.3 Occupancy Model

To accurately assess the spatial interactions between Chinese red pandas and various types of anthropogenic disturbances, we first identified the key environmental variables driving the distribution of each species using the optimal model derived from single‐season, single‐species occupancy modeling. Covariates associated with occupancy from the best model were the optimal covariates for each species. These covariates were then used in niche analysis for each species and incorporated into two‐species occupancy models. This approach ensured that the observed interspecific interactions were based on genuine ecological relationships between the species rather than environmental differences.

The single‐species occupancy model results (Table 3 and Table S6) revealed a Chinese red panda occupancy probability (ψ) of 0.540. Chinese red panda distribution showed a significant positive association only with elevation (ELE), increasing as elevation rose (*β* = 1.06, *p* = 0.034). In contrast, cattle and sheep exhibited occupancy rates of 0.298 and 0.037. Their distributions were co‐influenced by enhanced vegetation index (EVI), distance to water (DTW), and distance to residential area (DTR) (Table 4). Dogs and horses had occupancy rates of 0.037 and 0.075, respectively. No significant associations with any measured environmental factors were detected for either dogs or horses; consequently, only intercept‐only models were used for these species within the two‐species modeling framework.

**TABLE 3:** Optimal model and its occupancy probability.

Animal	Optimal models	$\hat{\psi }$
Chinese red panda	occu(~1 ~ ELE)	0.54
Cattle	occu(~1 ~ EVI + DTW + DTR)	0.30
Dog	occu(~1 ~ 1)	0.037
Horse	occu(~1 ~ 1)	0.075
Sheep	occu(~1 ~ EVI + DTW + DTR)	0.037

*Note:* $\hat{\psi }$ Occupancy probability.

Abbreviations: DTR, distance to residential area; DTW, distance to water; ELE, elevation; EVI, Enhanced Vegetation Index.

**TABLE 4:** Influence of environmental factors on species occupancy rate according to *β* coefficient (*p* < 0.05).

Environmental factor	Chinese red panda	Cattle	Dog	Horse	Sheep
ELE	1.06	—	—	—	—
ASP	—	—	—	—	—
SLP	—	—	—	—	—
EVI	—	−0.93	—	—	−2.36
DTD	—	—	—	—	—
DTW	—	1.71	—	—	3.80
DTR	—	−1.54	—	—	−3.98

Abbreviations: ASP, aspect; DTD, distance to road; DTR, distance to residential area; DTW, distance to water; ELE, elevation; EVI, Enhanced Vegetation Index; SLP, slope.

After controlling for the above environmental variables, the SIF derived from the two‐species occupancy models revealed the spatial relationships between the Chinese red panda and anthropogenic disturbance (Table 5). The results showed that the Chinese red panda exhibited significant spatial avoidance of cattle (SIF = 0.81, 95% CI: 0.72–0.93) and sheep (SIF = 0.60, 95% CI: 0.48–0.90), with all SIF values below 1. In contrast, the Chinese red panda showed significant positive spatial overlap with dogs (SIF = 1.53, 95% CI: 1.04–1.77) and horses (SIF = 1.80, 95% CI: 1.05–3.39), with all SIF values above 1. The 95% confidence intervals of all SIF estimates excluded 1.0, indicating that these spatial associations were not generated by chance. By integrating the single‐species and two‐species occupancy models, we found that the distributions of cattle and sheep were strongly associated with human activities, suggesting that high‐intensity anthropogenic disturbance drove the Chinese red panda to avoid cattle and sheep.

**TABLE 5:** Spatial interaction results between the dominant species A (anthropogenic disturbance) and the subordinate species B (Chinese red panda).

Animal	psiA	psiBA	psiBa	Mean SIF	SE	95% CI	Significance
Cattle	0.35	0.45	0.59	0.81	0.06	0.72–0.93	Yes
Dog	0.56	1	0.25	1.53	0.24	1.04–1.77	Yes
Horse	0.09	0.92	0.55	1.80	0.65	1.05–3.39	Yes
Sheep	0.048	0.39	0.56	0.60	0.12	0.48–0.90	Yes

### 4.4 Tweedie's Generalized Additive Model Analysis

**FIGURE 4:**
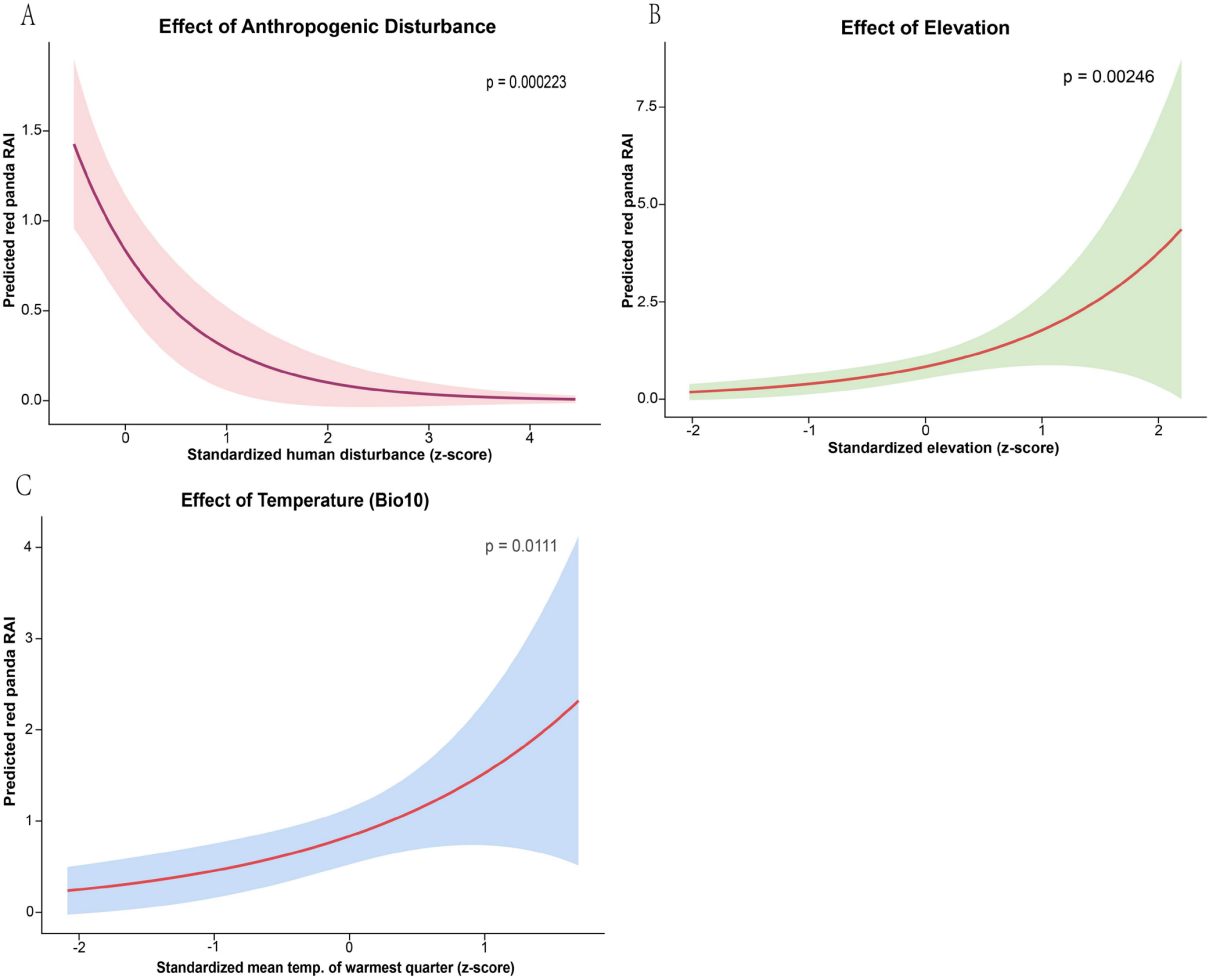
Partial dependence of the Chinese red panda RAI on environmental factors based on a Tweedie GAM. (A) Anthropogenic disturbance; (B) Elevation; (C) Mean Temperature of Warmest Quarter (Bio10). Solid lines represent the marginal mean RAI predicted by the model, and shaded areas indicate the 95% confidence intervals. The *p*‐value of each predictor is shown in the upper right corner of each panel. The results show that the RAI of the Chinese red panda decreased significantly with increasing anthropogenic disturbance intensity (*p* = 0.000223), and increased significantly with increasing elevation (*p* = 0.00246) and Mean Temperature of Warmest Quarter (*p* = 0.0111).

To quantify the direct effects of anthropogenic disturbances on the relative abundance of Chinese red pandas, we constructed a Tweedie GAM. Under control of topography, vegetation, and climatic variables, the model results (Table 6 and Table S7) revealed that anthropogenic disturbance intensity exhibited a negative effect on the RAI of Chinese red pandas (*β* = −1.055, *p* < 0.001) (Figure 4). Because *Z*‐score standardization (mean = 0, SD = 1) was applied prior to modeling, the regression coefficient for the relative abundance index of anthropogenic disturbance (*β* = −1.055) represents the effect of a one‐unit change in the standardized variable (i.e., one standard deviation change in the original disturbance intensity). Based on the log‐link function of the Tweedie GAM, the multiplicative change in the RAI is given by exp.(*β*). Accordingly, a one‐unit increase in the standardized disturbance intensity is predicted to reduce the relative abundance index of the Chinese red panda by (1 − exp.(−1.055)) × 100% ≈ 65.2%. The model also validated the significant positive effect of elevation (ELE) on Chinese red panda RAI (*β* = 0.753, *p* = 0.002) and the positive influence of the mean temperature of the warmest quarter (Bio10) (*β* = 0.603, *p* = 0.011) (Figure 4B,C). Other environmental and vegetation variables did not show significant effects.

**TABLE 6:** Results of Tweedie GAM regression for the relative abundance of the Chinese red panda in relation to anthropogenic disturbance, climatic, topographic, and vegetation factors.

Variable	Estimate	SE	*T* value	Pr(> |*t*|)	Significance
(Intercept)	0.330	0.523	0.631	0.530	
disturbance_RAI	−1.055	0.276	−3.816	0.000	***
ELE	0.753	0.243	3.099	0.002	**
Bio10	0.603	0.233	2.584	0.011	*
EVI	−0.179	0.212	−0.843	0.401	
DTD	0.129	0.363	0.355	0.723	
DTR	0.063	0.340	−0.187	0.852	
DTW	0.189	0.264	−0.715	0.476	
Evergreen broadleaf forest	0.326	2.114	0.154	0.878	
Shrubland	−1.074	1.434	−0.749	0.455	
Mixed evergreen‐deciduous broadleaf forest	−0.687	0.747	0.919	0.360	
Mixed coniferous‐broadleaf forest	0.510	0.553	0.923	0.358	
Coniferous forest	−0.650	0.627	−1.037	0.302	
Bio18	0.271	0.169	1.606	0.111	

*Note:* ASP, aspect; Bio10, Mean Temperature of Warmest Quarter; Bio18, Precipitation of Warmest Quarter; disturbance_RAI, relative abundance of anthropogenic disturbance.

Abbreviations: DTD, distance to road; DTR, distance to residential area; DTW, distance to water; ELE, elevation; EVI, Enhanced Vegetation Index; SLP, slope.

Signif.codes: ***p ≤ 001, **0.001 < *p* ≤ 0.01, *0.01 < *p* ≤ 0.05, ‘.’0.05 < *p* ≤ 0.1, ‘’*p* > 0.1.

In summary, this study employed multiple analytical approaches. Diel activity rhythm analysis revealed a high degree of temporal overlap between the Chinese red panda and anthropogenic disturbance (Δ4 = 0.85), indicating a potential encounter risk. The bivariate species distribution model directly confirmed that Chinese red pandas adopt a spatial avoidance strategy toward key disturbance sources (cattle and sheep), as evidenced by the calculated spatial interaction frequency (SIF < 1). Additionally, the single‐species distribution model and Tweedie GAM jointly demonstrated that both the occupancy probability and relative abundance of Chinese red pandas significantly increased with altitude, while anthropogenic disturbances exerted a direct negative impact on their abundance. Consequently, in response to intense anthropogenic disturbances, Chinese red pandas exhibited a coping strategy characterized by “passive temporal overlap, active spatial avoidance, and eventual concentration in higher‐altitude habitats.” However, the quantitative results from the Tweedie GAM explicitly showed that this spatial strategy was insufficient to completely mitigate the negative effects of disturbances, leading to a significant suppression of their population abundance.

## 5 Discussion

Our results indicated that livestock grazing was the primary form of anthropogenic disturbance affecting the Chinese red panda in the Sichuan Meigu Dafengding National Nature Reserve, with cattle exhibiting the highest occurrence frequency, followed by horses. The Chinese red panda adjusted its activity peak to avoid periods of high cattle activity, displaying a predominantly diurnal activity pattern with a peak around 12:00; nevertheless, its activity still showed significant temporal overlap with grazing disturbance. Seasonally, activity density was lowest in spring, only autumn exhibited a bimodal diel rhythm, and all other seasons were unimodal. Spatially, habitat use intensity increased with elevation; the Chinese red panda avoided cattle and sheep and showed a tendency to co‐occur with horses and dogs.

### 5.1 Adaptive Shifts in Diel Activity Rhythm in Response to Disturbance

We found that the Chinese red panda exhibited a predominantly diurnal activity pattern with some nocturnal activity, which is consistent with findings from the Mabian Dafengding Nature Reserve (Fu et al. 2020). Its activity peak was concentrated around 12:00, similar to the midday peak observed in the Chinese red panda in the Daxiangling Mountains (Feng et al. 2023), but different from the crepuscular bimodal rhythm reported in the Fengtongzhai Nature Reserve (Zhang et al. 2011). This difference may be attributed to two reasons. First, the Chinese red panda may adjust its activity pattern to reduce resource overlap with livestock (Yang et al. 2024). Second, in our study area, anthropogenic disturbance showed a high TRAI during crepuscular periods, which may have driven the Chinese red panda to shift its activity peak toward midday, resulting in a temporal niche shift.

As a specialized bamboo feeder, the Chinese red panda must consume large quantities of bamboo to compensate for its low nutritional quality, and therefore cannot reduce foraging time to cope with disturbance; instead, it tends to actively adjust its activity rhythm (Zhang et al. 2017). In areas of intensive human activity, the Chinese red panda often adopts a combined strategy of “vegetation cover + temporal activity shift” to reduce encounter risk (Bista et al. 2021). In this study, the high degree of temporal overlap in diel activity between the Chinese red panda and anthropogenic disturbance further confirms that the midday activity peak is not random, but rather represents a temporal niche shift away from the high‐disturbance crepuscular periods, allowing the species to meet basic needs such as foraging and movement while minimizing disturbance risk.

### 5.2 Seasonal Differences in Activity Density

At the seasonal scale, the activity density of the Chinese red panda in spring was significantly lower than in other seasons, and only autumn exhibited a bimodal diel activity rhythm, while the remaining seasons all displayed a unimodal pattern. Previous studies have shown that the Chinese red panda exhibits clear food selectivity, preferentially consuming young leaves and bamboo shoots of higher nutritional quality, and that the quality, distribution, and availability of food resources can influence its foraging strategy and spatial use (Zhang et al. 2009; Bista et al. 2023). The reduced activity in spring may be related to the increased availability of high‐quality food resources such as tender bamboo leaves and shoots, which reduces foraging effort. Camera‐trapping monitoring has also demonstrated that the Chinese red panda is more frequently active in autumn and winter (Fu et al. 2020). In this reserve, the staple bamboo species consumed by the Chinese red panda are *Yushania ailuropodina* and *Bashania fangiana* (Gu et al. 2022; Li et al. 2023). In summer, the active growth of *B. fangiana* at high elevations—a preferred bamboo species—drives an increase in the activity density of the Chinese red panda (Zeng et al. 2023). Autumn is a critical period for energy accumulation, during which the Chinese red panda supplements its diet with fruits of *Prunus* and *Rubus* species (Wei, Feng, Wang, and Li 1999), resulting in the highest activity density of the year. In winter, the activity density of the Chinese red panda remained relatively high, consistent with findings from the southern Yunling Mountains, where winter activity intensity was reported to be higher than in other seasons (Zhang et al. 2022). This may be associated with the increased energetic demands arising from reduced food availability and declining nutritional quality of bamboo during winter (Bista et al. 2025).

In summary, although the Chinese red panda exhibited adjustments in its diel activity rhythm, the high intensity and prolonged duration of grazing disturbance still resulted in a high degree of temporal overlap between the two activity patterns. This is consistent with the widespread adaptive pattern whereby wildlife adjust their activity timing to reduce disturbance exposure under high anthropogenic pressure (Hebblewhite and Merrill 2008).

### 5.3 Elevation Gradient Differentiation in Chinese Red Panda Habitat Utilization

Spatially, the intensity of habitat use by the Chinese red panda increased with elevation, which reflects avoidance of anthropogenic disturbance. Both ambient temperature and anthropogenic disturbance can drive the Chinese red panda to shift to higher elevations (Fu et al. 2020; Bista et al. 2025). High‐elevation areas provide cooler climates, and coniferous forests and alpine shrubs offer good concealment and shelter (Zhou et al. 2013). Meanwhile, results from the Third and Fourth National Giant Panda Surveys indicate increased grazing intensity in the Liangshan region (Li et al. 2019), and livestock severely damage bamboo forests through browsing and trampling, leading to significantly lower bamboo height and basal diameter in utilized areas compared to undisturbed areas (Li et al. 2017), forcing the Chinese red panda to shift to higher elevations. The Tweedie GAM further quantified this relationship: after controlling for vegetation, climate, and distance variables, anthropogenic disturbance had a significant negative effect on the relative abundance of the Chinese red panda, whereas elevation showed a positive effect, confirming that anthropogenic disturbance is a key driver of its upward elevational shift. Thus, the dependence of habitat selection on the elevation gradient reflects a dual strategy of “disturbance avoidance‐resource optimization” (Dong et al. 2021), and elevation acts as a critical spatial barrier insulating the Chinese red panda from anthropogenic disturbance.

### 5.4 Selective Spatial Response of Chinese Red Panda to Different Domestic Animals

The Chinese red panda exhibited differential spatial responses to different livestock species: it actively avoided cattle and sheep, while co‐occurring with horses and dogs. Cattle and sheep are typically herded in groups and their diet partially overlaps with that of the Chinese red panda (Hull et al. 2014). Furthermore, large‐scale grazing severely damages bamboo, the core food resource of the red panda (93% of activity sign plots contained bamboo) (Lama et al. 2020). The combined pressure of high competition and severe habitat degradation drives the Chinese red panda to adopt spatial avoidance. The two‐species occupancy models indicated that when cattle were absent, the site‐use probability of the Chinese red panda was 0.59, and when sheep were absent, it was 0.56, both significantly higher than the probabilities when they were present. Although horses also feed on bamboo, they usually move in small groups or solitarily, with large distances between individuals (> 10 m) (Hull et al. 2014; Borda et al. 2025), resulting in markedly weaker competitive pressure and habitat damage compared to cattle and sheep. Consequently, the Chinese red panda does not need to incur high avoidance costs and shows no spatial exclusion; the model showed that when horses were present, the site‐use probability of the Chinese red panda reached 0.92, significantly higher than when they were absent. Dogs possess strong searching abilities and can track small animals in complex environments (Karp 2020). The two‐species occupancy model revealed that when the Chinese red panda was present, the site‐use probability of dogs was 1. This does not indicate that the Chinese red panda does not avoid dogs; rather, it reflects the dogs' superior searching ability, which enables them to actively track and heavily overlap with the red panda's habitat sites. As a result, the red panda's spatial avoidance strategy becomes ineffective, leading to apparent overlap at the occupancy scale. If the Chinese red panda chose to avoid dogs by dispersing, it might increase the risk of mortality during movement (Bista et al. 2021).

### 5.5 Spatiotemporal Synergistic Responses of the Chinese Red Panda to Anthropogenic Disturbance

Taken together, the response of the Chinese red panda in the Meigu Dafengding Reserve to anthropogenic disturbance can be characterized as “temporal peak adjustment coupled with selective spatial avoidance.” Regression analysis showed that for each unit increase in anthropogenic disturbance, the relative abundance of the Chinese red panda decreased by approximately 65.2%, which was statistically significant, providing quantitative support for the driving mechanism behind its upward elevational shift. This synergistic strategy essentially represents a behavioral trade‐off under prolonged disturbance pressure, allowing the species to persist by shifting activity peaks and selectively avoiding certain disturbance sources, consistent with the risk‐disturbance hypothesis (Frid and Dill 2002). Through cross‐validation using diel activity rhythm analysis, two‐species occupancy models, and generalized additive models, this study reveals the combined spatiotemporal impacts of anthropogenic disturbance, thereby enhancing the robustness of our conclusions.

## 6 Conclusion

Our findings demonstrate that high‐intensity anthropogenic disturbance, particularly from domestic animal activities, poses significant pressure on Chinese red pandas in the Sichuan Meigu Dafengding National Nature Reserve, leading to adaptive adjustments at the behavioral ecology level. Temporally, the Chinese red pandas exhibited a predominantly diurnal activity pattern with a midday peak around 12:00, likely representing a temporal niche shift to reduce encounter risk with grazing disturbance during crepuscular hours. Seasonal activity intensity showed a distinct pattern of “low in spring, high in summer, autumn, and winter.” In spring, Chinese red pandas prioritized concealment due to disturbance pressure, whereas increased activity in summer, autumn, and winter was driven by resource demands and reduced disturbance. Despite temporal avoidance behavior, significant overlap persisted between Chinese red panda activity rhythms and anthropogenic disturbances throughout the year. Spatially, GAM analysis quantified a significant negative effect of anthropogenic disturbance on the Chinese red panda relative abundance index, while elevation exhibited a significant positive effect, confirming anthropogenic disturbance as a key driver of Chinese red panda migration to higher elevation areas. Consequently, Chinese red panda habitat use intensity increased with elevation, enabling avoidance of high‐intensity anthropogenic disturbance in low‐elevation zones, while higher elevations provided suitable bamboo resources and better vegetation cover for concealment. Moreover, Chinese red pandas exhibited selective responses to different domestic animals: active avoidance of cattle and sheep due to their herd behavior, high resource competition, and habitat degradation; and spatial co‐occurrence with horses, which are more dispersed and cause lower disturbance, as well as with dogs, owing to their strong olfactory tracking ability.

Therefore, to effectively conserve this endangered species, it is imperative to implement interventions that control the scale and spatial extent of anthropogenic disturbance within the Meigu Dafengding Nature Reserve, thereby mitigating its encroachment and impact on core Chinese red panda habitats. We recommend adopting a management strategy centered on spatiotemporally differentiated regulation.

Although the bamboo species variable did not reach statistical significance in the Tweedie GAM, bamboo is ecologically critical as the primary food source of the Chinese red panda, and its spatial distribution is vital for population persistence. Further analysis of bamboo species distribution (Figure S2) revealed that *Bashania fangiana*, which is highly preferred by the Chinese red panda, is mainly distributed in high‐elevation habitats, whereas *Yushania ailuropodina* is mostly concentrated in mid‐ and low‐elevation areas. Based on our findings, we propose the following conservation and management recommendations: strict elevational zoning comprising a “high‐elevation core conservation zone (> 3080 m, year‐round grazing prohibition), a mid‐elevation buffer zone (2855–3080 m, seasonal grazing restriction), and a low‐elevation management zone (< 2855 m, regulated grazing)” (Table S8 and Figure S3). Management efforts should prioritize preventing herd‐grazing cattle and sheep from entering vulnerable mid‐ and high‐elevation habitats and implementing tethering or confinement for associated livestock such as horses and dogs. During critical life‐history stages of the Chinese red panda, particularly the spring breeding period and peak diurnal activity hours, stricter restrictions on anthropogenic disturbance should be enforced. Meanwhile, community co‐management mechanisms and alternative livelihood projects should be actively promoted to alleviate conflicts between conservation and community development, and long‐term monitoring should be maintained to inform future adaptive management decisions.

### 6.1 Limitations of the Study

This study reveals the spatiotemporal responses of Chinese red pandas to anthropogenic disturbance, yet certain limitations should be acknowledged. First, the conclusions are based on short‐term observational data (from January 2023 to April 2024) from the Meigu Dafengding Nature Reserve, which is likely to limit their regional representativeness and temporal generalizability. Response strategies of Chinese red pandas to disturbance may vary across geographical regions or interannually. Second, the occupancy models were primarily constructed using data from the warm season and may not fully capture the dynamic interactions between disturbance and habitat use at an annual scale. Furthermore, the occupancy models and regression analyses did not fully encompass all potential influencing factors, which may interact with anthropogenic disturbance to collectively shape the spatiotemporal behavior of Chinese red pandas. Therefore, future research should expand the geographical scope, implement long‐term continuous monitoring, and integrate higher‐resolution environmental and biological factors to validate and refine the findings of this study, thereby constructing a more comprehensive and generalizable model of Chinese red panda responses to anthropogenic disturbance.

## Author Contributions

**Ying Fu:** formal analysis (equal), software (equal), visualization (equal), writing – original draft (equal), writing – review and editing (equal). **Minghua Chen:** project administration (equal), supervision (equal), writing – review and editing (equal). **Mengyi Duan:** data curation (equal), supervision (equal). **Bin Feng:** data curation (equal), supervision (equal). **Zhisong Yang:** data curation (equal), project administration (equal). **Ke He:** data curation (equal), project administration (equal), supervision (equal). **Hong Zhou:** conceptualization (equal), funding acquisition (equal), methodology (equal), supervision (equal), writing – review and editing (equal).

## Funding

This work was supported by National Natural Science Foundation of China, 32470538.

## Disclosure

This photograph of the Chinese red panda was taken by an infrared camera in the Sichuan Meigu Dafengding National Nature Reserve during this study. It is an original image that has not been previously published. The silhouette images of animals (dog, cattle, horse, and sheep) used in the graphical abstract are from OpenClipart (https://openclipart.org) and are dedicated to the public domain under the CC0 1.0 license.

## Conflicts of Interest

The authors declare no conflicts of interest.

## Supporting information


**Table S1:** Camera‐trap deployment in the Sichuan Meigu Dafengding National Nature Reserve.
**Table S2:** Relative abundance of Chinese red pandas and anthropogenic disturbance.
**Table S3:** Species domain model parameter description.
**Table S4:** Candidate models for the two‐species occupancy model.
**Table S5:** Kernel density estimation results.
**Table S6:** Single species occupancy model selection results (ΔAIC ≤ 2) for the Chinese red panda and anthropogenic disturbance (cattle, sheep, horses, and dogs).
**Table S7:** Model evaluation metrics for the Tweedie GAM.
**Table S8:** Management recommendations for different elevation zones based on the proportion of Chinese red panda occurrence.
**Figure S1:** Spearman correlation matrix of environmental predictor variables used in the occupancy models.
**Figure S2:** Preference of Chinese red pandas for different staple bamboo species based on the relative abundance index (RAI), and its relationship with the elevational distribution of five food bamboo species.
**Figure S3:** Conservation zoning based on elevational gradients.

## Data Availability

All original data and code have been deposited at Dryad and are publicly available as of the date of publication. DOI: https://doi.org/10.5061/dryad.n2z34tncq. Any additional information required to reanalyze the data reported in this paper is available from the lead contact upon request.
