# Supplementary material for: Temporal and Spatial Responses of Chinese Red Pandas to Anthropogenic Disturbance in Meigu Dafengding National Nature Reserve, Sichuan
Source: Ecol Evol. 2026 Aug 16;16(8):e74208. doi: 10.1002/ece3.74208 (PMC13477828; doi:10.1002/ece3.74208)
Supplement: Supplementary file 1 [file ECE3-16-e74208-s001.docx]

**SUPPLEMENTAL INFORMATION**

Table S1 Camera-trap deployment in the Sichuan Meigu Dafengding National Nature Reserve.

| Class | Variable | Number of cameras |
| --- | --- | --- |
| Elevation | 2,000<H≤2,500m | 47 |
|  | 2,500<H≤3,000m | 90 |
|  | 3,000<H≤3,500m | 49 |
|  | 3,500<H≤4,000m | 11 |
| Habitat type | Mixed evergreen-deciduous broadleaf forest | 19 |
|  | Mixed coniferous-broadleaf forest | 137 |
|  | Coniferous forest | 23 |
|  | Deciduous broadleaf forest | 16 |
|  | Evergreen broadleaf forest | 1 |
|  | Shrubland | 1 |

Table S2 Relative abundance of Chinese red pandas and anthropogenic disturbance.

| **Animal** | **Total number of valid photos** | **Relative abundance** |
| --- | --- | --- |
|  |  |  |
| Chinese red panda | 284 | RAI=（284/D）×1,000，D=30,041，result=9.453≈9.45 |
| Cattle | 737 | RAI=（737/D）×1,000，D=30,041，result=24.533≈24.53 |
| Sheep | 130 | RAI=（130/D）×1,000，D=30,041，result=4.327≈4.33 |
| Horse | 240 | RAI=（240/D）×1,000，D=30,041，result=7.989≈7.99 |
| Dog | 26 | RAI=（26/D）×1,000，D=30,041，result=0.865≈0.87 |
| Total | 1,417 |  |

Table S3 Species domain model parameter description.

| Parameter | Description |
| --- | --- |
| PsiA | The probability of species A using the site |
| PsiAB | The probability that species B uses a site when species A is present |
| PsiBa | The probability that species B uses a site when species A is absent |
| pA | The probability of detecting species A when species B is absent |
| pB | The probability of detecting object B when species A is absent |
| rA | The probability of detecting a species when both species are present |
| rBA | The probability of detecting species B when both species exist and species A is detected |
| rBa | The probability of detecting species B when both species exist and species A is not detected |

Table S4 Candidate models for the two-species occupancy model.

| Types of models | Hypothesis |
| --- | --- |
| psiA, psiB, pA, pB | Environmental variables and the presence of dominant species have no effect on the occupancy and detection of inferior species |
| psiA, psiB, pA, pB, rB | The presence of near dominant species has an effect on the detection of disadvantaged species |
| psiA, psiBA, psiBa, pA, pB | Only the presence of dominant species affects the detection of inferior species |
| psiA, psiBA, psiBa, pA, pB, rB | The presence of dominant species affects the occupancy and detection of inferior species |
| psiA(H), psiB(H), pA, pB | Only environmental variables affect the occupancy of inferior species |
| psiA(H), psiB(H), pA, pB, rB | The presence of dominant species mediated by environmental variables affects the detection of inferior species |
| psiA(H), psiBA(H), psiBa(H), pA, pB | The presence of dominant species mediated by environmental variables affects the occupancy of inferior species |
| psiA(H), psiBA(H), psiBa(H), pA, pB, rB | The presence of dominant species mediated by environmental variables affects both the occupancy and detection of inferior species. |

Table S5 Kernel density estimation results.

| **Season** | **Method** | **Overlap** | **CI_lower** | **CI_upper** | **P_value** | **N_panda** | **N_human** |
| --- | --- | --- | --- | --- | --- | --- | --- |
| Spring | Dhat4 | 0.838 | 0.767 | 0.903 | 0.015 | 97 | 541 |
| Summer | Dhat1 | 0.817 | 0.718 | 0.904 | 0.058 | 44 | 326 |
| Autumn | Dhat1 | 0.763 | 0.612 | 0.885 | 0.335 | 33 | 47 |
| Winter | Dhat4 | 0.780 | 0.723 | 0.873 | 0.003 | 110 | 219 |
| Annual | Dhat4 | 0.846 | 0.798 | 0.894 | 0 | 284 | 1,133 |

Table S6 Single species occupancy model selection results (ΔAIC ≤ 2) for the Chinese red panda and anthropogenic disturbance (cattle, sheep, horses, and dogs).

| Optimal Models | n | AIC | ΔAIC | AICwt | Occupancy_Rate |
| --- | --- | --- | --- | --- | --- |
| Chinese red panda |  |  |  |  |  |
| p(.), psi(ELE) | 161 | 820.658 | 0 | 0.133 | 0.540 |
| p(.), psi(ELE + DTD) | 161 | 821.465 | 0.807 | 0.089 | 0.559 |
| p(.), psi(ELE + DTW) | 161 | 821.470 | 0.812 | 0.088 | 0.534 |
| p(.), psi(ELE + DTR) | 161 | 821.797 | 1.139 | 0.075 | 0.553 |
| p(.), psi(ELE + SLP) | 161 | 821.907 | 1.25 | 0.071 | 0.547 |
| p(.), psi(ELE + EVI) | 161 | 822.594 | 1.936 | 0.050 | 0.540 |
| p(.), psi(ELE + SLP + DTD) | 161 | 822.650 | 1.992 | 0.049 | 0.553 |
| Cattle |  |  |  |  |  |
| p(.), psi(EVI + DTW + DTR) | 161 | 1290.443 | 0 | 0.304 | 0.298 |
| p(.), psi(ELE + EVI + DTW + DTR) | 161 | 1292.101 | 1.658 | 0.132 | 0.298 |
| p(.), psi(SLP + EVI + DTW + DTR) | 161 | 1292.229 | 1.786 | 0.124 | 0.298 |
| Sheep |  |  |  |  |  |
| p(.), psi(EVI + DTW + DTR) | 161 | 191.885 | 0 | 0.149 | 0.037 |
| p(.), psi(ELE + EVI + DTW + DTR) | 161 | 192.282 | 0.397 | 0.122 | 0.037 |
| p(.), psi(.) | 161 | 193.254 | 1.369 | 0.075 | 0.037 |
| p(.), psi(SLP + EVI + DTW + DTR) | 161 | 193.487 | 1.602 | 0.067 | 0.037 |
| p(.), psi(ELE + SLP + EVI + DTW + DTR) | 161 | 193.821 | 1.937 | 0.057 | 0.037 |
| Horse |  |  |  |  |  |
| p(.), psi(.) | 161 | 326.503 | 0 | 0.098 |  |
| p(.), psi(DTR) | 161 | 326.819 | 0.316 | 0.084 |  |
| p(.), psi(DTW) | 161 | 327.738 | 1.234 | 0.053 |  |
| p(.), psi(DTD) | 161 | 327.779 | 1.276 | 0.052 |  |
| p(.), psi(EVI) | 161 | 328.303 | 1.800 | 0.040 |  |
| p(.), psi(ELE) | 161 | 328.474 | 1.971 | 0.037 |  |
| p(.), psi(SLP) | 161 | 328.489 | 1.986 | 0.036 |  |
| Dog |  |  |  |  |  |
| p(.), psi(.) | 161 | 118.879 | 0 | 0.121 | 0.037 |
| p(.), psi(EVI) | 161 | 120.016 | 1.137 | 0.069 | 0.037 |
| p(.), psi(ELE) | 161 | 120.360 | 1.481 | 0.058 | 0.037 |
| p(.), psi(DTW) | 161 | 120.428 | 1.549 | 0.056 | 0.037 |
| p(.), psi(SLP) | 161 | 120.875 | 1.996 | 0.045 | 0.037 |

Table S7 Model evaluation metrics for the Tweedie GAM.

| **Metric** | **Value** |
| --- | --- |
| Deviance_Explained | 36.800 |
| Adjusted R-squared | 0.014 |
| REML | 205.420 |
| Scale_Estimate | 1.905 |
| Sample_Size | 126.000 |
| AIC | 436.250 |

Table S8 Management recommendations for different elevation zones based on the proportion of Chinese red panda occurrence.

| **Altitude zone** | **Elevation range (m)** | **Proportion of occurrence (%)** | **Management recommendations** |
| --- | --- | --- | --- |
| Low elevation | < 2,855 m | 33% | Standardized grazing |
| Mid-altitude | 2,855-3,080 m | 34% | Seasonal controls |
| High altitude | > 3,080 m | 33% | No grazing for the whole year |

Figure S1 Spearman correlation matrix of environmental predictor variables used in the occupancy models.

Figure S2 Preference of Chinese red pandas for different staple bamboo species based on the relative abundance index (RAI), and its relationship with the elevational distribution of five food bamboo species.

(A) Preference of Chinese red pandas for different bamboo species. Box plots showing the relative abundance index (RAI) of red pandas for five different bamboo species. The x-axis represents the bamboo species, and the y-axis indicates the RAI. The asterisks (*) denote that the red panda shows a significantly stronger preference for Bashania fangiana compared to the other bamboo species.

(B) Elevational distribution of different bamboo species. Box plots illustrating the elevational ranges of the five bamboo species. The x-axis represents the bamboo species, and the y-axis represents elevation (m). The asterisks indicate significant differences in elevational distributions among the bamboo species.

Figure S3 Conservation zoning based on elevational gradients.
